# Fanny pack transmission of carbapenem-resistant *Acinetobacter baumannii*

**DOI:** 10.1017/ash.2023.350

**Published:** 2023-09-29

**Authors:** Amber DelleFave, Juliana Mandarano, Nayef El-Daher

## Abstract

**Background:** Carbapenem-resistant *Acinetobacter baumannii* (CRAB) is a gram-negative coccobacillus that has garnered notoriety as a formidable cause of nosocomial infection with significant mortality. This organism poses a significant threat due to its multitude of resistance mechanisms and ability to endure within the environment. In the summer of 2022, a 350-bed acute-care hospital identified an outbreak of CRAB among critically ill patients in the intensive care unit (ICU) and intensive nursing care unit (INCU). Here, we report actions taken to contain the outbreak and to identify a common environmental source. **Methods:** In total, 7 nosocomial CRAB infection cases were identified by the infection prevention team between July and September 2022. A multidisciplinary team reviewed the cases using relevant medical history and available microbial susceptibilities. Clinical culture sites include 1 PICC tip, 1 urine sample, 1 peritoneal fluid samples, 5 wounds, and 1 sputum sample. Of 7 infections, 6 met the criteria for hospital onset, with an average time to infection from admission of 61 days. We quickly initiated universal contact precautions in the ICU and INCU for 6 weeks, enhanced daily cleaning of high-touch surfaces, provided staff and visitor education, conducted adenosine triphosphate (ATP) testing, collected observations, and performed selective environmental culturing based on observations. **Results:** In total, 71 environmental specimens were collected for culture. All were negative with the exception of 1 isolate obtained from the fanny pack of a wound-care nurse that was positive for CRAB. Also, 4 available patient isolates and the environmental isolate were sent to New York State Department of Health Wadsworth Center (NYSDOH Wadsworth) for genome sequencing, and relation to the same cluster was confirmed. Of 7 isolates, 6 were confirmed to express the *bla*OXA-23 resistance mechanism (1 was not available for testing). Subsequently, chart review identified that a wound-care nurse had had contact with all 7 patients within 30 days of their infections. **Conclusions:** After initiation of the described action plan, no further transmission was identified in the ICU or INCU. Real-time observation and environmental culturing was critical in identifying the epidemiological link, and this finding speaks to the ability of this organism to persist on a surface for a substantial length of time. Fanny pack use for transport of patient-care supplies was identified as a high-risk practice due to the inability to be properly disinfected between rooms and limited laundering. Fanny packs are no longer permitted in clinical spaces at this facility.

**Disclosures:** None

